# *ATAD3A*-related pontocerebellar hypoplasia: new patients and insights into phenotypic variability

**DOI:** 10.1186/s13023-023-02689-3

**Published:** 2023-04-24

**Authors:** Martina Skopkova, Hana Stufkova, Vibhuti Rambani, Viktor Stranecky, Katarina Brennerova, Miriam Kolnikova, Michaela Pietrzykova, Miloslav Karhanek, Lenka Noskova, Marketa Tesarova, Hana Hansikova, Daniela Gasperikova

**Affiliations:** 1 Department of Metabolic Disorders, Institute of Experimental Endocrinology, Biomedical Research Center SAS, Bratislava, Slovakia; 2 Laboratory for Study of Mitochondrial Disorders, Department of Paediatrics and Inherited Metabolic Disorders, First Faculty of Medicine, Charles University and General University Hospital in Prague, Prague, Czech Republic; 3 Research Unit for Rare Diseases, Department of Paediatrics and Inherited Metabolic Disorders, First Faculty of Medicine, Charles University and General University Hospital in Prague, Prague, Czech Republic; 4 Department of Paediatrics, Medical Faculty of Comenius University, National Institute of Children’s Diseases, Bratislava, Slovakia; 5 Department of Paediatric Neurology, Medical Faculty of Comenius University, National Institute of Children’s Diseases, Bratislava, Slovakia; 6 Department of Clinical Genetics, Institute of Medical Biology, Genetics and Clinical Genetics, Medical Faculty of Comenius University, University Hospital in Bratislava, Bratislava, Slovakia

**Keywords:** *ATAD3A*, Mitochondria, OXPHOS, Pontocerebellar hypoplasia

## Abstract

**Background:**

Pathogenic variants in the *ATAD3A* gene lead to a heterogenous clinical picture and severity ranging from recessive neonatal-lethal pontocerebellar hypoplasia through milder dominant Harel-Yoon syndrome up to, again, neonatal-lethal but dominant cardiomyopathy. The genetic diagnostics of *ATAD3A-*related disorders is also challenging due to three paralogous genes in the *ATAD3* locus, making it a difficult target for both sequencing and CNV analyses.

**Results:**

Here we report four individuals from two families with compound heterozygous p.Leu77Val and exon 3–4 deletion in the *ATAD3A* gene. One of these patients was characterized as having combined OXPHOS deficiency based on decreased complex IV activities, decreased complex IV, I, and V holoenzyme content, as well as decreased levels of COX2 and ATP5A subunits and decreased rate of mitochondrial proteosynthesis. All four reported patients shared a strikingly similar clinical picture to a previously reported patient with the p.Leu77Val variant in combination with a null allele. They presented with a less severe course of the disease and a longer lifespan than in the case of biallelic loss-of-function variants. This consistency of the phenotype in otherwise clinically heterogenous disorder led us to the hypothesis that the severity of the phenotype could depend on the severity of variant impact. To follow this rationale, we reviewed the published cases and sorted the recessive variants according to their impact predicted by their type and the severity of the disease in the patients.

**Conclusion:**

The clinical picture and severity of *ATAD3A*-related disorders are homogenous in patients sharing the same combinations of variants. This knowledge enables deduction of variant impact severity based on known cases and allows more accurate prognosis estimation, as well as a better understanding of the ATAD3A function.

**Supplementary Information:**

The online version contains supplementary material available at 10.1186/s13023-023-02689-3.

## Background

ATAD3A, the ATPase family AAA domain-containing protein 3, is a mitochondrial transmembrane protein that forms hexamers and is enriched in regions where mitochondria are in contact with endoplasmic reticulum [1, 2]. It belongs to the family of AAA + proteins (ATPases associated with various cellular activities) that comprises a wide range of molecular machines that utilize the energy from ATP hydrolysis to generate mechanical force and remodel their substrates [3]. The C-terminus of ATAD3A, located in the mitochondrial matrix, contains an ATPase domain and interacts with mitochondrial nucleoids [4, 5]. The transmembrane domains in the central part of the protein localize ATAD3A to span both the inner and outer mitochondrial membranes. The cytoplasm-oriented N-terminus interacts with other proteins with its proline-rich motif and two coil-coil domains [1]. ATAD3A is implicated in diverse cellular processes including mitochondrial structure [6], dynamics [7], nucleoid organization [4], mitophagy [8], and cholesterol metabolism [9, 10].

The human *ATAD3A* gene (MANE select v0.95 transcript NM_001170535.3, ENST00000378756.8 (ATAD3A-203), 2,481 nt, 586 amino acids) is located on the distal part of the short arm of chromosome 1 (1p36.33), together with its two paralogues, *ATAD3B* and *ATAD3C*, which emerged in hominids during evolution through segmental duplication [11]. The sequence homology of this region predisposes it to rearrangements, and, indeed, the most frequent mutations in the ATAD3 locus are structural variations including deletions, duplications, and rearrangements [10, 12–14]. Moreover, 18 different small nucleotide variants have been found to date and include recessive loss of function (nonsense, frameshift), recessive hypomorphic (missense), as well as dominant negative (missense) mutations [14–18].

Impairment of the *ATAD3A* gene function results in a spectrum of disorders. Biallelic loss-of-function pathogenic variants lead to **neonatal lethal pontocerebellar hypoplasia, hypotonia, and respiratory insufficiency **(MIM#618810, PHRINL) with encephalopathy, corneal clouding, and mild cardiomyopathy [14]. Recessive hypomorphic variants lead to milder phenotypes with a prolonged lifespan up to adulthood [17]. Monoallelic dominant missense pathogenic variants lead to delayed development, mild intellectual disability, hypotonia with spasticity, peripheral neuropathy, optic atrophy, and mild cardiomyopathy known as **Harel-Yoon syndrome** (MIM#617183) [14]. Finally, monoallelic dominant duplications in the ATAD3 gene cluster locus (containing *ATAD3A*, *ATAD3B*, and *ATAD3C* genes) lead to multisystemic **chromosome 1p.36.33 duplication syndrome, ATAD3 gene cluster** (MIM#618815) [12, 13] with severe cardiomyopathy, hypotonia and corneal clouding and variable neurological findings including seizures and white-matter, basal ganglia, thalamus or cerebellar changes on brain imaging.

Sixty-five patients have been described in the literature since the discovery of *ATAD3A* as a disease-causing gene in 2016 [14]. From this number, 29 patients had the dominant and 36 patients had the recessive form of the disease.

Here we report two additional families, each with two affected children with *ATAD3A* mutations, and provide a summary of published cases and variants. Furthermore, we propose that the severity of clinical picture correlates with the predicted severity of *ATAD3A* variants. This genotype-phenotype correlation allows for an estimation of prognosis in patients to be identified in the future.

## Results

### Clinical picture of the patients

**Fig. 1:**
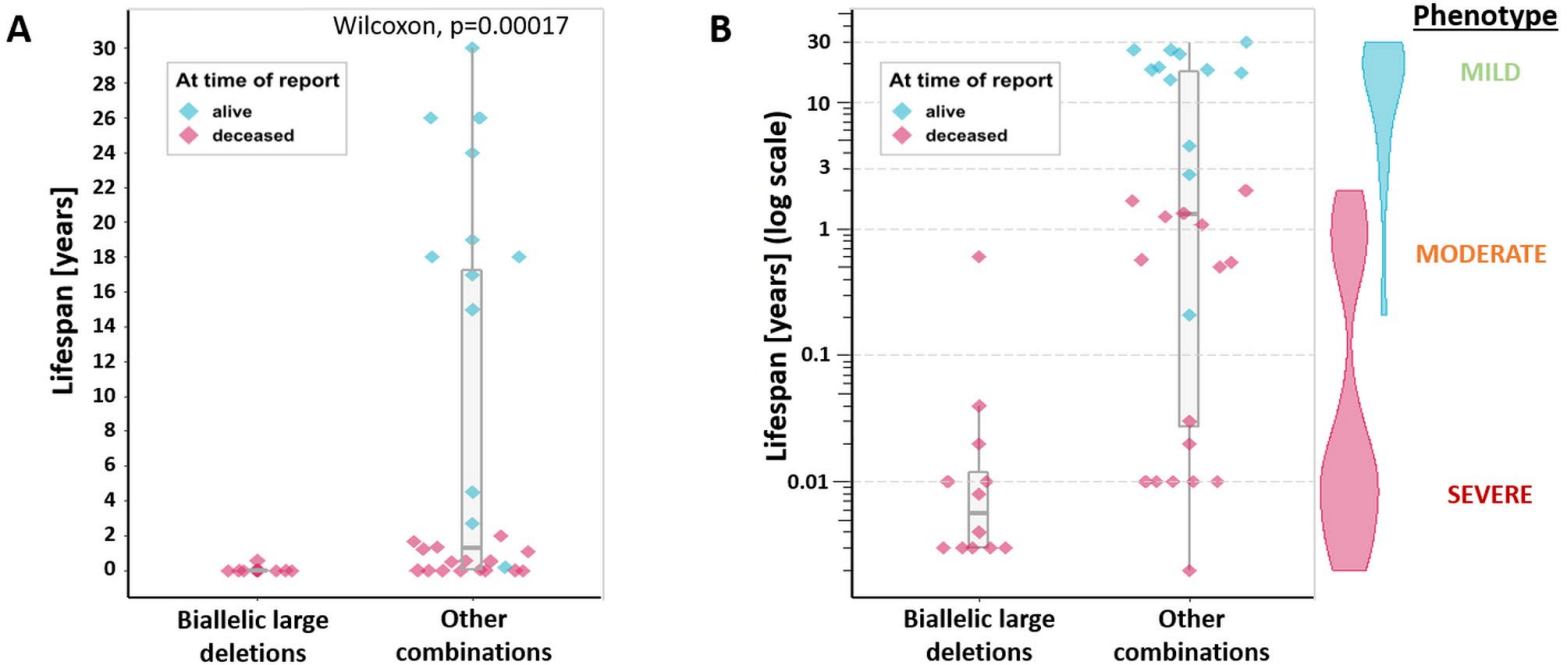
Pedigrees and identification of variants in ATAD3A gene **(A)** Pedigree of Family 1, visualisation of the c.229C>G, p.(Leu77Val) in WES data, and long-range PCR confirming deletion of exons 3 and 4; **(B)** Pedigree of Family 2, visualisation of the c.229C>G, p.(Leu77Val) and copy-number variation analysis from WES data, and long-range PCR confirming deletion of exons 3 and 4; **(C)** The read covering the breakpoint in the deletion of exons 3 and 4 in WES data of Patient 3

In this study, we describe two families with two affected siblings each (Fig. 1A and B). All four affected patients died at the age of 13–20 months. The clinical course was similar in all four patients, with rapid progression. All four children were born after an uneventful pregnancy. No prenatal brain or heart anomalies and no IUGR were reported in both children. The common features included congenital stridor, and cataract, severe hypotonia with hyporeflexia, feeding difficulties and failure to thrive, leukodystrophy, and cerebral or cerebellar atrophy with an enlarged ventricular system that developed during the first months of life. In Family 1, seizures and cardiomyopathy developed during life in both affected siblings; in Family 2, axonal demyelinating polyneuropathy was present in the examined Patient 3. The complete phenotype of Patient 4 is not known as the parents declined extensive investigation. The patients did not have a marked increase of lactate or alanine in plasma or cerebrospinal fluid; only moderate 3-methylglutaconic aciduria was present. Neurodegeneration and cataracts were the leading symptoms considered in clinical differential diagnostics.

### Genetic testing and classification of variants

Compound heterozygous variants c.229C>G (p.Leu77Val) and a deletion of exons 3 and 4 in the *ATAD3A* gene were identified in all four affected patients. Parents and healthy siblings were tested and the variants co-segregated with the disease in both families (Fig. 1A and B).

The variant c.229C>G (p.Leu77Val) (rs138594222) in exon 2 has already been reported in one patient in combination with the whole gene deletion [17]. Thus, a total of five patients have been reported with this variant found *in trans* with a clearly pathogenic loss-of-function variant [PM3_very strong]. The variant is present in ClinVar (ID 432628) in six patients as a variant of unknown significance. The overall population frequency in gnomAD v3.1.1. is 0.0006466 (44/68,046 alleles, no homozygotes), which doesn’t allow for an automatic granting of PM2_supp. Another variant in the same position, p.Leu77Arg, has been reported [19], but the Grantham score for Leu to Arg substitution is larger than that for Leu to Val (102 vs. 32, respectively); therefore, the PM5 criterium is not met. In silico tools do not predict a damaging impact either (REVEL score 0.09). However, a functional study performed on *Drosophila* confirmed increased mitophagy, loss of mitochondria, and aberrant cristae [17] [PS3_supp]. The phenotype of the patients is in line with the clinical picture described in *ATAD3A* patients [PP4]. Considering all this information, the variant is classified as likely pathogenic.

The second variant found *in trans* was the deletion spanning exons 3 and 4 identified using a CNV analysis of WES data. It was detected in one patient only, but the visual inspection of the coverage in this region suggested the presence of this deletion also in the mother and the affected sibling (Fig. 1B). Long-range PCR confirmed its presence in all affected patients and subsequent sequencing of the breakpoint confirmed that it is the same deletion variant as reported previously in two siblings in Yap et al. [17] [PM3_very strong]. This variant is not present in the population database gnomAD SVs v2.1 [PM2_supp]. The deletion of exons 3 and 4 is predicted to result in an in-frame deletion of 162 nucleotides (encoding 54 amino acids) forming the CC1 domain involved in the oligomerization of the ATAD3A protein and its binding to the outer mitochondrial membrane [PVS1_strong]. Together with the patients’ phenotype in line with the clinical picture of ATAD3A-related disease [PP4], we classified this variant as pathogenic.

The families were not aware of any relatedness and came from different parts of Slovakia. A comparison of phased VCF files for the individuals from the families did not show any indices of a larger common haplotype (Supplementary Tables 1, Additional file 1). The common sequence in the allele with the deletion variant was less than 268 kbp with only two shared informative polymorphisms, a very common rs7418389 (MAF 0.48) and a novel 5’ UTR variant chr1-1447592-C-G (GRCh37) (MAF 0). The common sequence around the c.299C>G variant is only 159 kbp and there are no shared polymorphisms in this region.

### Patient 1 biochemistry

**Fig. 2:**
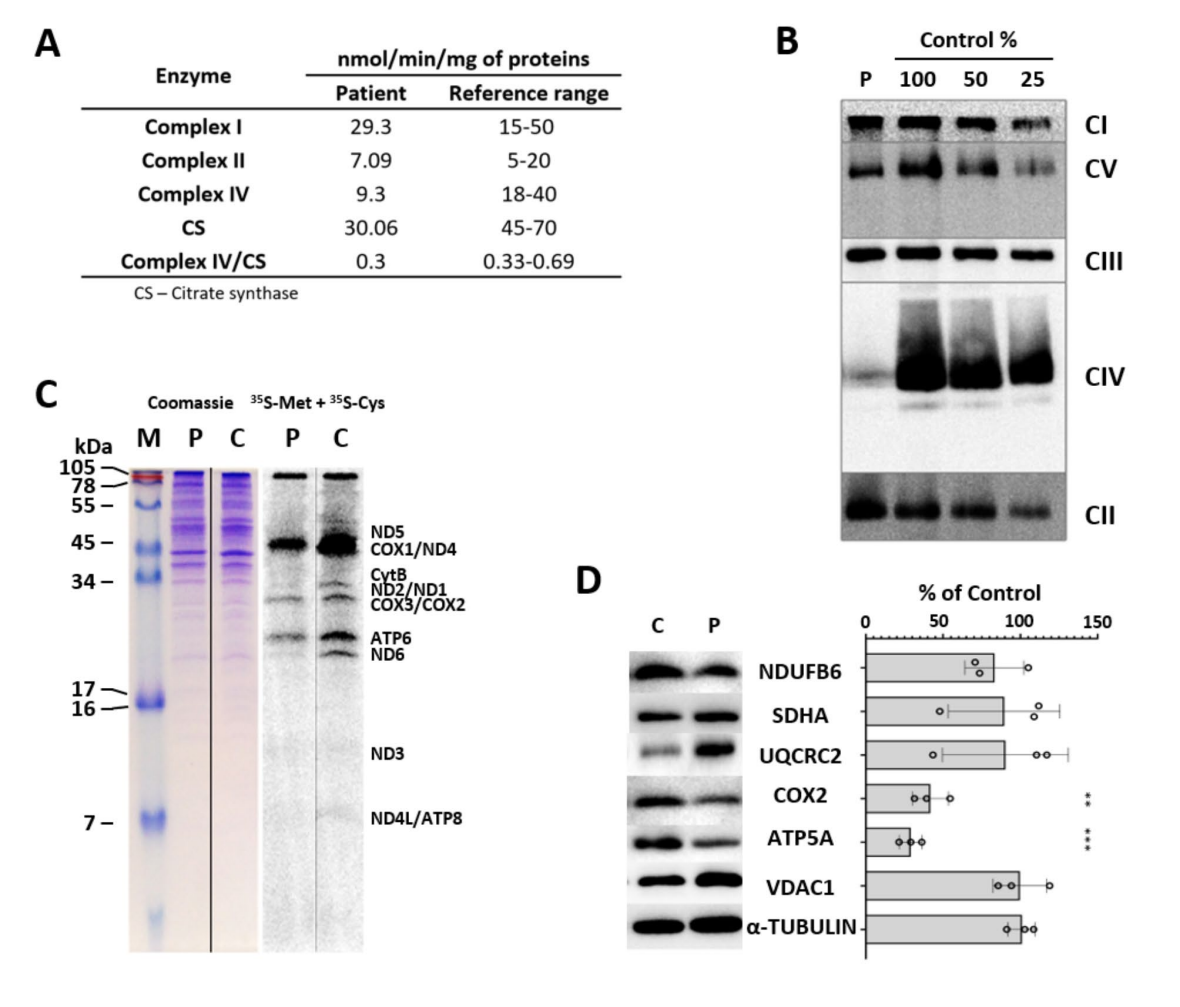
Analysis of oxidative phosphorylation system complexes in Patient 1 **(A)** Activities of respiratory chain complexes and citrate synthase in cultured fibroblasts in Patient 1; **(B)** BN-PAGE/WB/Immunodetection showed decreased levels of complex IV, complex I and complex V holoenezyme in muscle mitochondria (CI-NDUFA9, CII-SDHA, CIII-UQCRC2, CIV-COX1, CV ATP5A). **(C)** Mitochondrial proteosynthesis rate measured using metabolic labelling with ^35^ S-Met + ^35^ S-Cys was only slightly decreased to 84% of the control; **(D)** In fibroblasts, significantly decreased levels of COX2 and ATP5A, complex IV and complex V subunits, respectively, were noted. Results of three independent analyses were used and one representative blot is presented. (** *p* < 0.01, *** *p* < 0.001) C – Control, M – Marker, P - Patient

Testing of the respiratory complex enzymatic activities in cultured fibroblasts from Patient 1 revealed decreased complex IV activity (Fig. 2A). A severely decreased level of complex IV holoenzyme and a mildly reduced amount of complex I and complex V holoenzymes were seen in muscle mitochondria (Fig. 2B). In fibroblasts, decreased levels of subunits COX2, and ATP5A were noted (Fig. 2D). The mitochondrial proteosynthesis rate was only slightly decreased to 84% of the control (Fig. 2C).

### Assessment of lifespan in reported cases with recessive variants

**Fig. 3:**
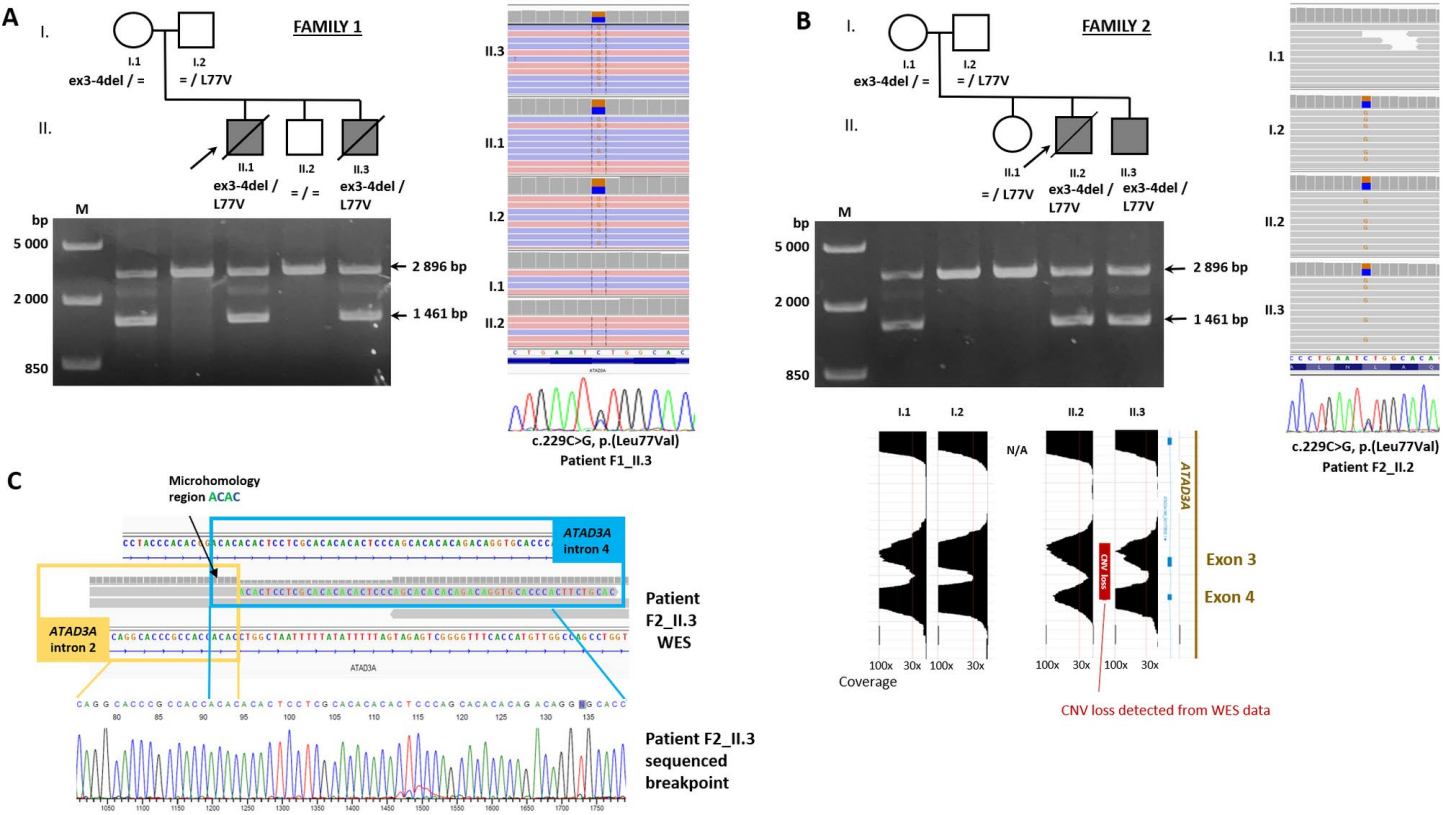
Lifespan of patients with biallelic ATAD3 locus large deletions compared to other patients. **(A)** The two groups showed significantly different lifespan (Wilcoxon test, p = 0.00017) with **(B)** a tri-modal distribution of the values visible on the logarithmic scale and density violin plots of living and deceased patients

We divided the cohort of 40 reported cases with recessive variants into two subsets, patients with biallelic large deletions extending into the *ATAD3A* gene and patients with all other combinations of pathogenic variants. The two groups showed significantly different lifespan (Wilcoxon test, p = 0.00017) (Fig. 3A). A tri-modal distribution of the lifespan values is visible on the logarithmic scale (Fig. 3B). The most severe form is neonatal lethal, which we labeled as the “severe form”. Cases in which the patient survived the neonatal period but died in early childhood were labeled as the “moderate form”, and, finally, patients with later onset and living into adulthood were labeled as the “mild form”. The details of the reviewed patients and variants are given in Supplementary Tables 2 and 3, respectively (Additional file 1).

## Discussion

Since 2016, when the mutations in the *ATAD3A* gene were identified as causative in the first group of patients [14], a total of 69 patients from 52 families (Supplementary Tables 2, Additional file 1, including those in this study, with various types of variants (Supplementary Tables 3, Additional file 1) have been reported [10, 12, 13, 15, 16, 18, 19]. In this study, we describe 4 new patients and show that the severity of the phenotype of patients with the recessive form of *ATAD3A*-linked disorder is relative to the impact of the variants.

The four patients from the two described families had compound heterozygous variants c.299C>G (p.Val77Leu) and deletion of exons 3 and 4. The comparison of their genotyping data showed that the alleles carrying mutations do not come from a recent common ancestor. The c.299C>G variant is found in fairly the same frequency in multiple subpopulations in gnomAD. This speaks against a local founder effect for this variant, as well. The exon 3–4 deletion was created most probably due to microhomology in intron 2 and 4 and might have arisen multiple times independently due to non-allelic homologous recombination. That is also supposed in case of large deletions which have been found in patients from all around the world and various ancestries [20]. However, the presence of the 5’ UTR variant chr1-1447592-C-G, which is not found in population databases, in linkage with the exon3-4 deletion suggests that these two alleles may have arisen from the same ancestor, but probably not very recently.

Three main types of *ATAD3A*-related disorders can be observed. Two of them are autosomal dominant – (i) Harel-Yoon syndrome (p.Arg528Trp variant in 5 cases) [14] or milder hereditary spastic paraplegia (p.Gly355Asp in 2 patients from 1 family) [18], where the patients survive into adulthood; and (ii) autosomal dominant perinatal lethal cardiomyopathy (4 types of ATAD3 locus duplications in 22 cases) with variable neurological phenotype and strong cardiac impairment with neonatal lethality [12, 13].

The third, iii) autosomal recessive form, leads to pontocerebellar hypoplasia with a range of severity from neonatal lethal to patients living into adulthood. Large deletions encompassing the *ATAD3* region or resulting in a generation of a fusion gene between *ATAD3B* and *ATAD3A* are the most common variants. Due to the high sequence homology, the fusion cDNA carries only several missense variants, but it is expressed from the *ATAD3B* promotor on a very low level [10, 12, 17]. Thus far, various combinations of different recessive point mutations, large deletions, or rearrangements have been found in 40 patients from 25 families (including this study).

The clinical picture and severity of the four patients described herein matched strikingly with that of the patient from Family 3 depicted in [17] (Supplementary Tables 4, Additional file 1). All five patients carried a loss of function variant on one allele and the variant p.Leu77Val on the second allele. This consistency of the phenotype in patients sharing the same variants in an otherwise heterogeneous disorder led us to the assumption that the severity of the phenotype could depend more on the type and severity of the pathogenic variants than on the influence of external factors or other genetic modifiers (which of course can still play a role in individual cases).

As proof of this concept, we gathered all patients with biallelic large deletions variants and compared their lifespan with the lifespan of all other patients with recessive *ATAD3A* mutations together (Fig. 3A). The large deletions comprising part of the *ATAD3A* gene including its promoter were proved experimentally to result in a fusion gene expression under the *ATAD3B* or *ATAD3C* promoter, depending on the deletion. This decreases significantly the *ATAD3A* expression in adult tissues [10, 12]. If the idea that the severity of phenotype correlates with the severity of the variant impact was correct, we would expect these patients to have the most severe form of the disorder. And indeed, 11 of the 12 patients with biallelic deletions deceased in the first two weeks of life. One of them died at 7 months, however, this patient was incapable of breathing without support, therefore the increased lifespan appears to have been caused by the duration of the intervention, rather than being classified as a milder phenotype [20]. The fact that the two groups showed significantly different lifespan (Fig. 3A) proves that abolished *ATAD3A* expression dramatically decreases the lifespan of the patients. This claim can be extended to the variants, where there is a large scientific consensus that they are null alleles - premature termination codons and frameshift and canonical splice site variants leading to nonsense-mediated decay (here p.Gln164*, Val381Glyfs*17 and His472Thrfs*14).

Next, when we use the life span of the patients as the marker of the disease severity, we can divide the patient’s phenotype into three groups – severe, moderate and mild, according to the tri-modal distribution evident from Fig. 3B. Considering previously mentioned points, the most logical explanation of the existence of milder phenotypes is that these patients carry at least one allele with partially preserved activity.

Therefore, we suggest that the impact of the missense and single amino acid deletion variants can be inferred from the severity of the disease, taking into consideration the variant on the other allele, as follows. The variants that were neonatal lethal in the homozygous state or in combination with a null variant are labeled “high impact” (p.Phe50Leu, p.Leu77Arg, p.Leu406Arg). Variants found either compound heterozygous with a null variant (p.Leu77Val, p.Gly236Val) or homozygous (c.384+3A>G, p.Arg327Pro) in patients with the moderate severity are labeled as “medium impact”. And finally, variants found homozygous or in combination with a null variant in patients living into adolescence and adulthood (p.Thr53Ile, p.Arg170Trp, p.Lys568del) or with a milder course of the disease with normal mental development in childhood (p.Thr84Met) are considered as “low impact”. Two variants, p.Trp537Arg and p.Arg503Profs*11, were found in a compound heterozygous state in two siblings with the mild form in this combination only. As the latter is predicted to escape nonsense-mediated decay, it is currently not possible to predict their individual impact. Supplementary Tables 5, Additional file 1, sums up the sorting process. The lifespan of the patients grouped according to the deduced combinations of alleles is presented in Supplementary Fig. 1, Additional file 1.

**Fig. 4:**
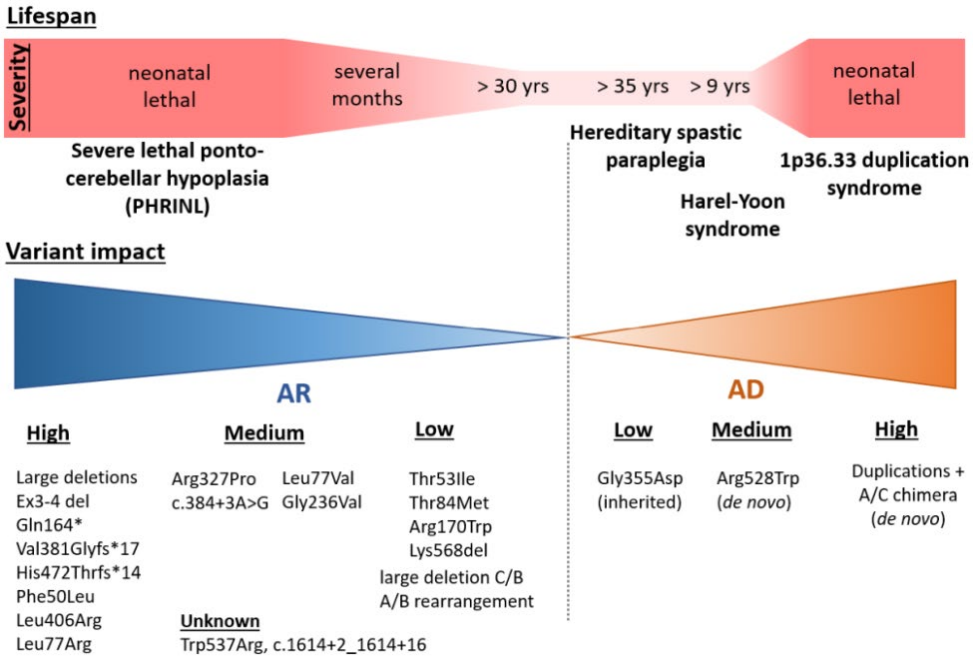
Summary of predicted impact of variants in the ATAD3A gene. The given *ATAD3A*-related diseases are reduced to a short description and represent complex multisystem phenotypes. The impact of variants was inferred from the patient’s lifespan and the nature of a variant on the second allele

The residual activity of the medium and the low-impact hypomorphic variants is most probably a continuum, and various combinations of alleles will result in a continuum of disease severity. Nevertheless, we believe that our stratification (summarized in Fig. 4) will help in better prognosis estimation in further patients to be diagnosed and may also be helpful in future structure-function studies.

The results of the functional studies for some of these variants performed by Yap et al. in *Drosophila* knock-outs [17] showed that only variants homologous to human p.Leu77Val (labeled “medium impact”) and p.Arg170Trp (“low impact”) rescued the developmental lethality caused by *dAtad3a* gene loss, indicating some residual functionality. Nevertheless, other variants tested – p.Phe50Leu (“high impact”), p.Arg327Pro (“medium impact”), p.Gly236Val (“medium impact”), and p.Lys568del (“low impact”) – did not rescue the developmental lethality of the *dAtad3a* knock-out. This suggests that *Drosophila* is a good conservative model for *ATAD3A* variant testing, but the capability of a variant to rescue the lethality in *Drosophila* doesn’t have to correlate with the outcomes of the patients.

Peralta et al. [15] suggested that mutations affecting the C-terminal region with the ATPase domain located in the matrix could be more severe than those affecting the N-terminal region containing two transmembrane domains and the coiled-coil domains involved in ATAD3A interactions with other protein partners outside mitochondria. However, since then several recessive variants have been discovered in both regions, and the genotype-phenotype correlation doesn’t seem to be dependent on the position Supplementary Fig. 2, Additional file 1).

Nevertheless, the two dominant missense variants discovered so far, p.Arg528Trp and p.Gly355Asp causing HAYOS and spastic paraplegia, respectively, are both located at the ATP-binding site. Glycine 355 is directly in the Walker A motif and arginine 528 is located in the Sensor-II motif, opposite the glycine 355 in the conserved pocket [18]. Moreover, the third type of dominant mutations – various duplications found in the patients with neonatal lethal cardiomyopathy – result in the expression of a fusion gene *ATAD3A*-*ATAD3C*, where the N-terminal part corresponds to the *3 A* gene, whereas the C-terminal ATPase domain comes from the *3 C* pseudogene. The chimeric *A/C* gene contains multiple variants, including seven variants in the ATPase domain [12, 13]. Among them, Arg466Cys deserves special interest, as arginine 466 is conserved in all multimeric AAA-domain containing ATPases and functions as an arginine finger, a trans-acting residue that binds to the γ-phosphate of ATP in the neighboring monomer [3]. Therefore, it is probable, that the dominant variants act through the incorporation of an ATPase-deficient monomer into ATAD3A hexamers [13]. Different clinical consequences of these three dominant variants, mainly the strong cardiac involvement in the case of A/C chimera expression, however, remain unexplained and underline the involvement of ATAD3A in a number of processes that can differ in various tissues.

Furthermore, the impairment of the respiratory chain in the studied patients seems to be variable, as well. Deficiency of complex IV activity was seen in the fibroblasts of our Patient 1 (Fig. 2A), but in the muscle mitochondria, we detected decreased content not only of complex IV but of complexes I and V, as well (Fig. 2B). The - significantly decreased levels of COX2 and ATP5A, complex IV and complex V subunits, respectively, seen in the patient’s fibroblasts (Fig. 2D) and the decreased mitochondrial proteosynthesis (Fig. 2C) confirm a combined OXPHOS deficiency in this patient. In other published cases with biallelic variants, decreased activities of complexes I, III, or IV were reported, and a decrease in all complexes on the protein level has also been described, similarly to our results [10]. Combined OXPHOS deficiency agrees with what would be expected in the case of impairment of the ATAD3A function. However, multiple patients with biallelic large deletions and the severe phenotype showed only borderline or even normal activities in fibroblasts or in muscle and liver [10], and patients with mild phenotype also had either decreased CI and CIV complexes [10] or normal activities in fibroblasts or muscle cells [14]. This shows that in the case of recessive *ATAD3A*-related disorder variable complexes can be affected and that impairment of the respiratory chain is not necessarily detected in all tissues or in all patients. Interestingly, in the case of the dominant A/C chimera causing perinatal lethal cardiomyopathy, markedly decreased complex I activity occurred selectively in cardiac muscle [12], underlying the importance of the role of ATAD3A in cardiomyocytes. It is thus possible that OXPHOS activities are more affected in the cells of the nervous system in the case of the recessive variants, where the neurological symptoms are predominant, but this has not been studied yet.

*ATAD3A*-related disorders are most probably underdiagnosed due to the difficult-to-analyze chromosomal region containing three gene paralogs. Mitochondriopathies are genetically and phenotypically heterogeneous, therefore NGS techniques are the preferred method of choice for DNA diagnostics. However, copy-number variation analysis from WES data is still tricky and the presence of unidentified CNVs may often be a cause of negative analysis results in patients. When a targeted analysis was performed, *ATAD3A* locus mutations were the fourth most common nuclear-encoded cause of mitochondriopathy in a group of > 500 pediatric-onset cases in Australia and New Zealand [12]. Furthermore, variants such as p.Leu77Val can be filtered out during analysis based on location in the segmental duplication, higher population frequency or negative *in silico* predictions.

Moreover, variants in the *ATAD3A* gene might also play a modifier role in other diseases. We published a case of a patient with Leigh syndrome caused by mutations in the *SURF1* gene several years ago, Patient 1 in [21]. This patient showed an extremely severe clinical course, which was not typical for the *SURF1*-related Leigh disease. Interestingly, it has now been revealed, that this patient also carried the above-mentioned p.Leu77Val variant on a single allele, in addition to biallelic pathogenic variants in the *SURF1* gene. The modifying effect of *ATAD3A* variants would, certainly, have to be supported by further studies and other cases.

## Conclusions

The most pronounced symptoms in our four patients sharing the same combination of variants were neurodegeneration and cataracts. Despite the geographic proximity of the two described families, no recent common ancestry was shown for the studied c.299C>G (p.Leu77Val) end exon 3 and 4 deletion variants. The clinical picture and severity of symptoms in patients with *ATAD3A*-related disorders are homogenous in patients sharing the same combination of variants. The respiratory chain activities, on the other side, show greater variability among tissues and patients with the same ATAD3A variants. This knowledge can enable faster diagnosis, more accurate prognosis estimation, as well as a better understanding of the ATAD3A function.

## Patients and methods

### Patients

Family 1 – healthy parents of Caucasian origin from Slovakia with two affected sons from the first and the third pregnancy and one unaffected son from the second pregnancy. The pregnancies with affected children were uneventful.

#### Patient 1

The boy was born full-term (40th gestational week) from the first pregnancy, with a birth weight of 3,280 g and a length of 50 cm, Apgar score of 6/8, and moderate birth asphyxia. Congenital stridor, bilateral cataracts, strabismus and asymmetry of cerebral ventricles were noted. The patient was operated on the cataracts under general anesthesia at the age of 5 months. During pre-operative tests, a heart murmur was noted, but the patient was hemodynamically compensated. Mild or starting cardiomyopathy however cannot be excluded. Hypotonic syndrome with hyporeflexia and developmental delay manifested during the first months of life. Hypertrophic cardiomyopathy was found during hospitalization in the 7th month. Magnetic resonance imaging revealed leukoencephalopathy and cortico-subcortical cerebral and cerebellar atrophy with enlarged ventricles. Percutaneous endoscopic gastrostomy was carried out due to failure to thrive in the 7th month. Tonic-clonic seizures occurred in the 8th month and the child decompensated during febrility leading to respiratory failure. He died in 13 months. During life, metabolic screening revealed increased lactate only once in plasma, otherwise only intermittently in urine, without metabolites indicating energetic disbalance; lactate in the cerebrospinal fluid was normal, 3-methylglutaconic acid was increased (73.3 µmol/mmol of creatinine). During life, thiamin, pyridoxin, coenzyme Q10 and carnitine were supplemented to the patient to support energetic metabolism, as mitochondriopathy was suspected due to multisystemic involvement and progressive clinical course. Patient 1 corresponds to patient II.1 in Family 1 (Fig. 1A).

#### Patient 2

The brother of Patient 1 from the 3rd pregnancy was born full-term (38 gw) with a weight of 2,890 g and a length of 50 cm. Screening brain ultrasonography showed perivascular and basal ganglia calcifications. After an illness in 3 months, bilateral cataracts and convergent strabismus were noticed. Axial hypotonus with acral hypertonia, central coordination impairment, and ptosis were observed upon neurological examination. EMG findings were without neurogenic or myogenic lesions. The patient’s clinical course deteriorated during febrile illness at the age of 11 months, when convulsions and tonic-clonic seizures occurred with respiratory insufficiency, acidosis, plasma lactate up to 3.3 mmol/L, and mild 3-methylglutaconic aciduria. Further examination showed hypertrophic cardiomyopathy and mild brain atrophy with an enlarged ventricular system. The patient was fed by gastrostomy and a tracheostomy was performed due to laryngomalacia and stridor. The patient died at the age of 15 months. During life outside the crisis, lactate was normal in plasma and liquor, alanine increased in plasma, and mildly increased excretion of Krebs cycle metabolites, lactate (110 µmol/mmol of creatinine), 3-OH-butyrate, methylmalonate, and from the age of 6 months increased 3-methylglutaconate (30.4–82.9 µmol/mmol of creatinine) was detected in urine. The treatment during life included thiamin, pyridoxin, coenzyme Q10 and carnitine. Patient 2 corresponds to patient II.3 in Family 1 (Fig. 1A).

Family 2 – healthy parents of Caucasian origin from Slovakia with one healthy daughter and two affected sons from the second and the third uneventful pregnancies.

#### Patient 3

The boy was born full-term (38 gw) with a weight of 3,070 g and length of 50 cm, Apgar score of 10/10. In the 3rd month of age, failure to thrive was noted, as well as progressive hypotrophy, hypotonia, mixed quadriparesis with hyperkinetic movements of extremities, laryngeal stridor, and cataracts. In 10 months, postnatal hypotrophy due to feeding difficulties (weight under the 3rd percentile) and psychomotor developmental delay, convergent strabismus and decreased nerve conduction velocity on electromyography were added. The cardiologic examination was negative. In the 17th month, PEG had to be introduced due to the failure to thrive. Brain MRI showed bilateral periventricular leukoencephalopathy and cerebellar hypotrophy with enlarged ventricular system, and progress to axonal demyelinating polyneuropathy with hyporeflexia was seen on EMG. Metabolic investigation during life revealed only mild 3-methylglutaconic aciduria (increased twice, the highest value 86 µmol/mmol of creatinine, otherwise under 25 µmol/mmol of creatinine). The treatment during life included thiamin, pyridoxin and coenzyme Q10. The patient died at the age of 20 months. Patient 3 corresponds to patient II.2 in Family 2 (Fig. 1B).

#### Patient 4

The brother of Patient 3 was born full-term (40 gw) from the 3rd pregnancy with a weight of 3,460 g and a length of 50 cm. In the 3rd month of life, cataract and central hypotonia with acral hypertonia were noticed. The child started to fail to thrive, and bulbar syndrome developed in the 6th month, leading to the need of nasogastric feeding at the age of 12 months. Loss of weight and hypotonia progressed, and hyperkinetic movements of extremities and oral automatisms were added. Brain MRI and further investigations (EMG, cardiological and biochemical follow-up) were declined by the parents, as the diagnosis with poor prognosis had already been established. The treatment included thiamin, pyridoxin and coenzyme Q10. Laboratory parameters were normal except for persistent mild 3-methylglutaconic aciduria (< 20 µmol/mmol of creatinine). The patient died at the age of 17 months. Patient 4 corresponds to patient II.3 in Family 2 (Fig. 1B).

## Methods

### Genetic analysis

All genetic analyses were performed from genomic DNA extracted from peripheral blood using standard procedures. Written informed consent for genetic testing was obtained from all participating subjects.

The whole exome sequencing (WES) was performed at the Research Unit for Rare Diseases, Department of Pediatrics and Inherited Metabolic Disorders, First Faculty of Medicine of the Charles University in Prague (SeqCap EZ Exome Probes v3.0, Roche) – family 1, or as a service in Theragen, South Korea (SureSelect XT V6, Agilent) – family 2 and Patient 2 from family 1. Analysis of family 1 (the parents, Patient 1, and the healthy sibling) was performed by an in-house bioinformatic pipeline. Reads were aligned to the hg19 reference genome using Novoalign version 3.02.13 (Novocraft) with default parameters. After genome alignment, conversion of SAM format to BAM and duplicate removal was performed using Picard Tools (2.20.8). The Genome Analysis Toolkit, GATK (3.8) [22] was used for local realignment around indels, base recalibration, variant recalibration, and variant calling. Analysis of family 2 – FASTQ files for parents and two affected children were loaded into the Congenica analysis platform, within which Sentieon DNAseq® pipeline was used for alignment to GRCh38 and variant calling, ExomeDepth tool for CNV calling, and HPO-based Exomiser tool was used for variant prioritization. Patient 2 from family 1 was identified retrospectively by search of an in-house Gemini SQLite database [23] of more than 200 rare-disease cases analyzed with WES in Biomedical Research Center SAS. GATK (3.8) was used for haplotype calling and VCF phasing for both families.

Candidate variants were confirmed by long-range PCR (according to [17] for exon 3–4 deletion) and by Sanger sequencing (primer sequences and sequencing strategy to overcome sequence homology issues in Supplementary Tables 6, Additional file 1).

The chromosomal and *ATAD3A* coding positions refer to the GRCh38 and NM_001170535.3 reference sequence, respectively. The variant classification was done according to ACMG guidelines [24] with modifications for PVS1 according to [25], PS3 according to [26], and for PM2 and PM3 criteria according to ClinGen recommendations (https://clinicalgenome.org/working-groups/sequence-variant-interpretation/).

### Mitochondria isolation

Post-mortem muscle (*m.gastrocnemius*) obtained at the autopsy of Patient 1 was snap frozen in liquid nitrogen within 12 h after death. Muscle mitochondria were isolated according to standard differential centrifugation procedures [27] in a buffer containing 150mM KCl, 50mM Tris/HCl, 2mM EDTA, and 2 µg/ml aprotinin (pH 7.5) at 4 °C [28]. The homogenate was centrifuged for 10 min at 4 °C and 600 *g*, the postnuclear supernatant was filtered through a 100 μm nylon membrane, and mitochondria were sedimented by centrifugation for 10 min at 4 °C and 10,000 *g*. The mitochondrial pellet was washed by centrifugation and resuspended to a final protein concentration of 20–25 mg/ml. Protein concentration was determined by the Lowry method [29], and the isolated mitochondria were stored at -80 °C.

The primary skin fibroblasts of Patient 1 were established from skin obtained during the muscle autopsy procedure. Fibroblasts were cultured in high-glucose DMEM medium (Dulbecco’s Modified Eagle Medium; PanBiotech) supplemented with 10% (v/v) fetal bovine serum (GE Healthcare) and Antibiotic-Antimycotic (Biosera) at 37 °C in 5% CO_2_ atmosphere.

### Spectrophotometry

The activities of respiratory chain complexes in fibroblasts were measured according to [30] in the cell suspension. The activity of citrate synthase (CS, EC 2.3.3.1), serving as the control enzyme to avoid assay variability, was measured according to [31]. Protein concentrations were measured by the Lowry method [29].

### Electrophoresis and immunoblot analysis

To analyze the steady-state levels of mitochondrial oxidative phosphorylation system (OXPHOS) protein complexes, Blue Native Polyacrylamide Gel Electrophoresis (BN-PAGE) of n-dodecyl β-d-maltoside (DDM) solubilized isolated muscle mitochondria was used (final ratio 4 DDM/ mg protein) [32]. Protein concentration was determined by BCA assay (Thermo Scientific™, Waltham, MA, USA). A total of 2.5–10 µg of protein was loaded per lane and separated by 6–15% polyacrylamide gradient gels (MinProtean® 3 system; Bio-Rad, Hercules, CA, USA). Serial dilutions of the control sample (25-100%) were loaded on the same gels.

Tricine Sodium Dodecyl Sulfate–Polyacrylamide gel electrophoresis (SDS-PAGE) was carried out under standard conditions with 12% polyacrylamide, 0.1% (w/v) SDS gels. Fibroblast cell homogenates were incubated for 20 min on ice with RIPA buffer (50 mM Tris/HCl (pH 7.4), 150 mM NaCl, 1 mM PMSF, 1 mM EDTA, 1% Triton X-100 and 0.1% SDS (v/v), 1% (v/v) Protease Inhibitor Cocktail (PIC, Sigma-Aldrich)) and centrifuged at 51 000 g for 25 min, 4 °C. Samples were dissociated in 50 mM Tris/HCl (pH 6.8), 12% (v/v) glycerol, 4% SDS, 2% (v/v) 2-mercaptoethanol, and 0,01% (w/v) Bromphenol Blue for 30 min at 37 °C. 5–10 µg of protein was loaded in each lane. Serial dilutions of a control sample (50–100%) were loaded on the same gels. The signal quantification of individual antibodies proteins was performed using Quantity one software. The results from three independent experiments were expressed as percentages related to 100% control. The data were statistically analyzed using the analysis of variance (ANOVA) and graphs were generated in GraphPad Prism 9.3.1.

BN-PAGE and SDS-PAGE gels were transferred onto Immobilon-P PVDF Membrane (Millipore, Burlington, MA, USA) by semi-dry electroblotting using the Hoefer semi-dry transfer unit (Hoefer, Harvard Bioscience, Holliston, MA, USA). Primary detection of blots was performed using mouse monoclonal antibodies against NDUFA9 (ab14713, Abcam, dilution 1:2,000), NDUFB6 (ab 110,244, Abcam, dilution 1:4,000), SDHA (ab14715, Abcam, dilution 1:20,000), UQCRC2 (ab14745, Abcam, dilution 1:20,000), COX1 (ab14705, Abcam, dilution 1:10,000), COX2 (ab110258, Abcam, dilution 1:10,000), ATP5A (ab110273, Abcam, dilution 1:4 000), VDAC1 (ab14734, Abcam, dilution 1:2 000), and rabbit monoclonal antibody α-Tubulin (2125 S, Cell Signaling, dilution 1:2,000). Blots were incubated with primary antibodies in TBS, 0.1% (v/v) Tween 20, and 1% non-fat dried milk for 2 h. The secondary detection was carried out with anti-Mouse IgG (whole molecule)–Peroxidase antibody produced in goat (A8924, Sigma-Aldrich, dilution 1:2,000) or anti-Rabbit IgG (whole molecule) – Peroxidase antibody produced in goat (A0454, Sigma-Aldrich, dilution 1:2,000) in TBS, 0.1% Tween 20, and 1% non-fat dried milk for 1 h. The blots were visualized with SuperSignal™ West Femto Maximum Sensitivity Substrate (TermoFisher Scientific) using Syngene G:Box (Syngene) and analyzed by Quantity One software (Bio-Rad).

### Metabolic labeling

To study the synthesis of mtDNA-encoded proteins, we essentially employed the method of in vivo metabolic labeling by ^35^S-methionine + ^35^S-cysteine as described previously [33], using Express [^35^S] Protein Labeling Mix (PerkinElmer, USA). Cells were labeled in DMEM, without methionine and cysteine, and the radioactive labeling mix with 200 µCi/mL for 2 h. Afterward, 20 µL of the mixture of “cold” methionine and cysteine (non-labeled Met and Cys) was added into each well (final concentration 250 µM) and incubated for a further 15 min. Then, cells were washed three times with phosphate-buffered saline PBS containing 250 µM cold methionine and 250 µM cold cysteine. Emetine dihydrochloride hydrate was present in the medium throughout the experiment as an inhibitor of cytoplasmic proteosynthesis at a concentration of 100 µg/ml. Cellular pellets were later used for SDS-PAGE analysis. The 20 µg protein aliquots of each sample were separated by 16% gel. Following the electrophoretic run, the gel was fixed in a staining solution (40% (v/v) methanol, 8% (v/v) acetic acid, 0.05% (w/w) Coomassie Brilliant Blue R-250) and dried. Radioactivity was detected by exposing the gel to Storage Phosphor Screen BAS-IP SR 2025 E for 7 days (GE Healthcare), which was then scanned by FX Molecular Imager (Bio-Rad).

### Statistical assessment of the lifespan

The plots and statistical analysis of patients’ lifespan was done using the open-source R Statistical Software package [34]. The data were checked for normality using the Shapiro-Wilcoxon normality test using the base (4.0.5) package and the means were compared using the two-sided non-parametric Wilcoxon rank-sum test with continuity correction using the stats (4.0.5) package for R. The plots were generated using the ggplot2 and ggExtra packages.

## Electronic supplementary material

Below is the link to the electronic supplementary material.

**Additional file 1**: Supplementary Tables 1 – Haplotypes of the alleles present in the patients in the region surrounding the *ATAD3A* gene. Supplementary Tables 2 – Summary of reported patients with *ATAD3A* mutations. Supplementary Tables 3 – Summary of reported variants in the *ATAD3A* gene. Supplementary Tables 4 – Clinical picture of patients carrying p.Leu77Val variant in addition to a null allele. Supplementary Tables 5 – Sorting of variants according to their impact. Supplementary Fig. 1: Lifespan of patients with deduced combinations of variants. Supplementary Fig. 2: The variants plotted across the ATAD3A protein scheme. Supplementary Tables 6 – Sequences of primers used for long-range PCR and for breakpoint and exon 2 sequencing.

## Data Availability

All data are available from the corresponding author upon reasonable request.
